# Supplementary figures and images for: HDAC-6 inhibition ameliorates the early neuropathology in a mouse model of Krabbe disease
Source: Front Mol Neurosci. 2023 Jul 27;16:1231659. doi: 10.3389/fnmol.2023.1231659 (PMC10426153; doi:10.3389/fnmol.2023.1231659)

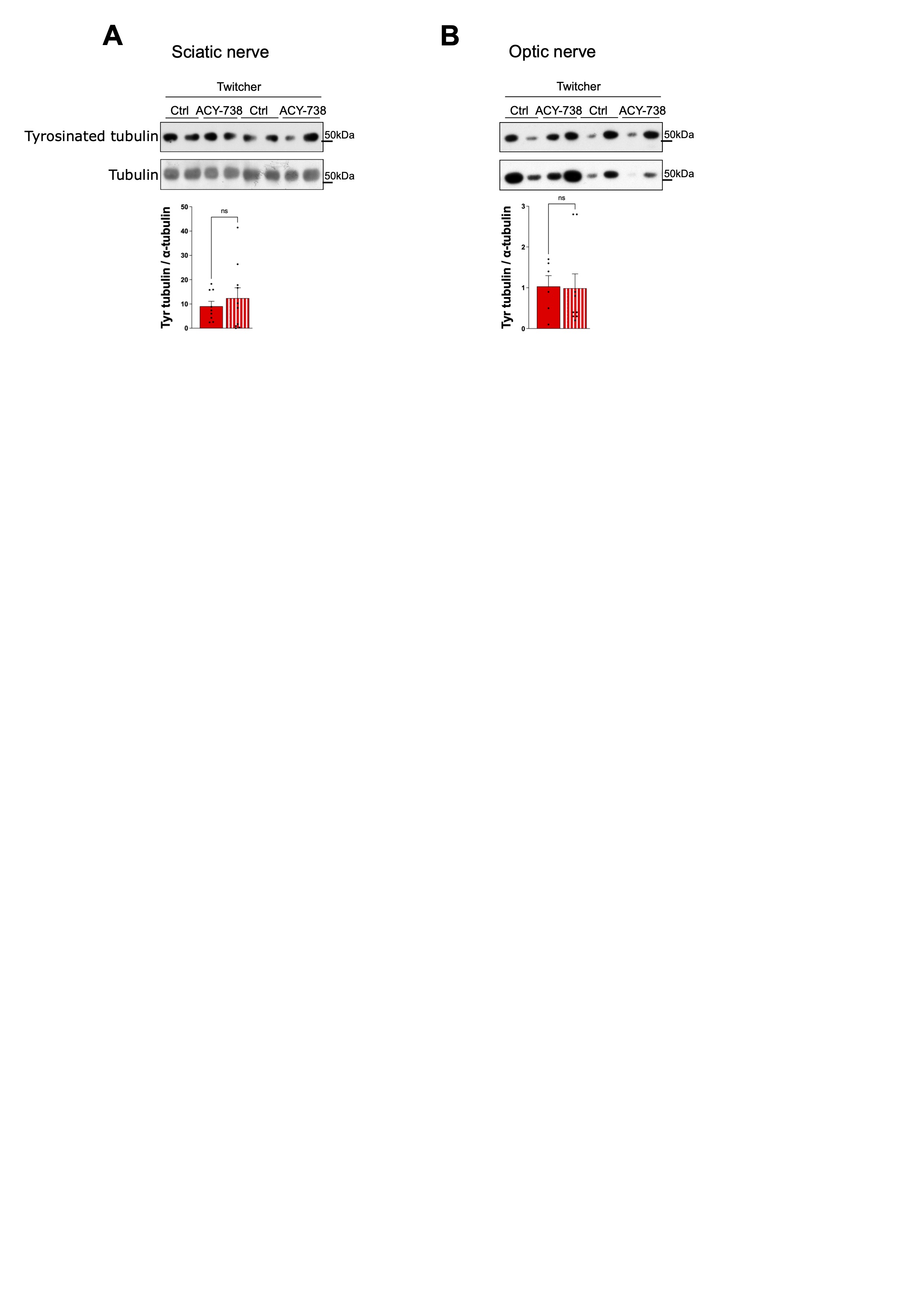

Supplement: SUPPLEMENTARY FIGURE S1 — Western blot analysis of tissues from WT and Twitcher mice treated with ACY-738 from P0 until P9. Western blot analysis of tyrosinated tubulin and α-tubulin in (A) sciatic nerves and (B) optic nerves of P9 Twitcher mice untreated or treated with ACY-738 (upper panels) and respective quantifications (lower panels). Data represent mean ± SEM (*p < 0.05, two-tailed unpaired t-test) of n = 6-8 control Twitcher and n = 9 ACY-738-treated Twitcher. [file Image_1.jpeg]
